# Comparative transcriptome analysis of the different tissues between the cultivated and wild tomato

**DOI:** 10.1371/journal.pone.0172411

**Published:** 2017-03-09

**Authors:** Qi Dai, Lili Geng, Minjia Lu, Weibo Jin, Xuying Nan, Ping-an He, Yuhua Yao

**Affiliations:** College of Life Sciences, Zhejiang Sci-Tech University, Hangzhou, China; Huazhong University of Science and Technology, CHINA

## Abstract

Although domesticated tomato is cultivated by wild tomato, there are a lot of differences between cultivated tomato and wild tomato, such as shape, physiological function and life history. Many studies show that wild tomato has better salt resistance and drought resistance. In addition to, domesticated tomato’s fruit is bigger and has more nutritious than wild tomato. The different features are closely related to differentially expressed genes. We identified 126 up-regulated differentially expressed genes and 87 down-regulated differentially expressed genes in cultivated tomato and wild tomato by RNA-Seq. These differentially expressed genes may be associated with salt resistance, drought resistance and fruit nutrition. These differentially expressed genes also further highlight the large-scale reconstruction between wild and cultivated species. In this paper, we mainly study GO enrichment analysis and pathway analysis of the differentially expressed genes. After GO and pathway enrichment analysis, a set of significantly enriched GO annotations and pathways were identified for the differentially expressed genes. What’s more, we also identified long non-coding RNAs and mRNAs in the two species and analyzed its essential features. In addition to, we construct a co-expression network of long non-coding RNAs and mRNAs, and annotate mRNAs associated with long non-coding RNAs as target genes, and speculate the regulation function of long non-coding RNAs. In total, our results reveal the effects of artificial and natural selection on tomato’s transcript, providing scientific basis for tomato’s research in the future.

## Introduction

Tomato [1, 2] is one of the most scientifically investigated vegetables because of its high commercial importance [3]. In addition, the tomato is highly perishable, and post-harvesting losses reach 25 to 50%. In tropical countries, there is a loss of 20–50% from harvesting to consumption [4–6]. What is more, the water content of tomato fruit is very high, and the water content is as high as 93–95% [7]. In addition, it is low in calories and rich in vitamins A, C and E and minerals, such as calcium, potassium, phosphorus. Tomato is the first in terms of contribution in the diet in a rank of 10 kinds of vitamins and minerals [8]. Brazil is the largest tomato producer in South America, followed by Chile and Argentina. Northeastern region (Pernambuco and Bahia states) accounted for 46% of the production [9]. In recent years, the consumer’s demand for tomato products has increased, and it is increasing rapidly in domestic and international markets with major portion of it being used for preparation of convenience food. Because tomato is rich in antioxidants, so it can be considered important source of carotenoids (lycopene), ascorbic acid and phenolic compounds. In addition, the heat increases the bioavailability of lycopene, which is better absorbed by the body when the tomato is cooked, thus, ideal for the consumption of tomato sauces and soups. The industrialization process of the tomato shows that the preparation of sauces, ketchup and others does not destroy lycopene.

In some cases, the genetic basis of phenotype associated and domestication have been examined, most notably in maize, rice, and tomato [9–11]. It is clear that the difference of domesticated and wild phenotype is from a small number of genetic loci. Some studies show that the transcriptional level is changing all the time during the domestic process. For example, the recent studies have indicated that the transcriptional network of maize has changed a lot during the domestic process. Although changes in gene expression or network topology are important, there is a lack of expression between domesticated and wild tomato during the domestic process.

In recent years, researchers study tomato from various aspects. Tomato is a kind of important domesticated specie. Meanwhile, Tomato is a member of a complex of 13 interfertile species that occupy a wide range of habitats in South America. In the growth process of tomato, there are 998 predicted transcription factors which belong to 62 families and account for about 2.87% of 34727 genes in tomato genome. In 2012, the tomato has been sequenced successfully. The decoding genome of tomato identifies about 34727 genes, while 97.4% genes have located on the chromosomes accurately [12]. The difference of salt tolerance is compared in wild tomato and cultivated tomato. Tal and Smith‘s study [13] shows that there is positive correlation between the growth of callus and the whole plant. So, plant and callus are used to study the salt resistance under the condition of salt stress in some sweet soil crops, such as tomato. Dry weight of wild plant was higher than cultivated plant and the water content of wild tomato is also higher than cultivated tomato. There is difference between wild tomato and cultivated tomato. Compared with wheat, corn, potato, sugar and sunflower, tomato is a moderate salt sensitive plant. Salt resistance is regulated by the specific stage, and every stage is regulated by multiple genes and environment.

Tomato originated in the tropics is a typical thermophilic crop. Temperature is an important factor in process of tomato’s growth and plays an important role in northern China. Low temperature is one of interferential factors for tomato’s production [14]. Jiang et al [15] mainly study wild and cultivated tomato’s tolerance of low temperature and illustrate low temperature response mechanism in different varieties. The phenotypic diversity of wild tomato is a good artificial and natural selection system for domesticated tomato. However, the transcriptional information of tomato is still unclear. Our study aims at comparing wild tomato and cultivated tomato based on their transcriptional information. In consequence, wild tomato has better salt resistance and drought resistance, and cultivated tomato’s fruit is bigger, and has rich nutrition [16, 17].

Our research data is mainly from Koenig D’s experimental data [18]. In Koenig D’s study, he mainly focused on several aspects: the large-scale alteration of the light response co-expression network between wild and cultivated accessions; characterization of sequence diversity in wild and cultivated tomato; evidence for positive selection in wild and cultivated tomato; divergence in gene expression in wild and cultivated tomato; analysis of selective pressures on gene expression, evolution of the tissue-specific expression in Solanum pennellii and Solanum lycopersicum, effect of introgression on the transcriptome of domesticated tomato; expression divergence correlates with phenotypic differences among wild and cultivated accessions.

Based on Koenig D’s research, we carried out the much more extensive and intensive research for tomato. We analyzed domesticated and wild tomato’s transcriptome to identify the diversity of gene expression sequences based on natural and artificial selection. Our data contains cultivated tomato (S. lycopersicum) and wild tomato (S. pennellii). The reason of selecting wild tomato is that it is widely used for genetic donation in the process of improving cultivated tomato. Our analysis provides sufficient evidence for tomato’s evolution and presents significantly differences between artificial and natural selection.

## Materials and methods

### Data resource

Transcribed sequences from cultivated and wild tomato were mainly collected from the Sequence Read Archive (SRA) database [19], as follow (Table 1). Data available in the Sequence Read Archive from tomato included 14 samples from 7 distinct tissues (root, stem, leaf, flower, fruit, seedling and vegetative) encompassing a total of 62676 transcripts.

**Table 1:** The sequence number of the research data.

Tissue	cultivated tomato (*Solanum lycopersicum*)	wild tomato (*Solanum pennellii*)
Root	SRR786526 SRR786527 SRR786528 SRR786543 SRR786544	SRR786558 SRR786559 SRR786572 SRR786573
Stem	SRR786531 SRR786532 SRR786547 SRR786548	SRR786562 SRR786563 SRR786576 SRR786577
Leaf	SRR786524 SRR786525 SRR786540 SRR786541 SRR786542	SRR786556 SRR786557 SRR786570 SRR786571
Floral	SRR786520 SRR786521 SRR786535 SRR786536 SRR786537	SRR786552 SRR786553 SRR786566 SRR786567
Fruit	SRR786522 SRR786523 SRR786538 SRR786539	SRR786554 SRR786555 SRR786568 SRR786569
Seeding	SRR786507 SRR786508 SRR786509 SRR786529 SRR786530 SRR786545 SRR786546	SRR786510 SRR786511 SRR786512 SRR786513 SRR786560 SRR786561 SRR786574 SRR786575
Vegetative	SRR786533 SRR786534 SRR786549 SRR786550 SRR786551	SRR786564 SRR786565 SRR786578 SRR786579 SRR786580

### Data analysis

For the RNA-seq data, the quality of data was evaluated by the FastQC software [20], and we retained reads that contained more than 95% bases and the bases’ quality score is 20.

This program, Tophat-Cufflinks [21], could process a large number of read fragments based on RNA-Seq [22–23]. Transcripts selected could be processed as follows: (1) Aligning RNA-Seq reads to the reference genome. It was a core step in the analysis workflows, and we used Tophat [24] to align RNA-Seq reads to the genome. (2) Assembling transcripts. We used Cufflinks [25] packages to assemble transcripts. Frist, cufflinks assembled transcripts. Then, cuffmerge merged two or more transcript assemblies. Third, cuffdiff identified differentially expressed genes, transcripts and detected differential splicing and promoter. Besides, the analysis of differentially expressed genes was also conducted by FPKM.

### Identification of lncRNAs and DGEs

Differentially expressed genes (DEGs) were selected according to the threshold: |log2fold change| ≥ 1.00. False discovery rate (FDR) was used to correct the P values and genes with FDR < 0.05 were considered as significantly DGEs. We used in-house Python script to select applicable genes as DGEs. The false discovery rate (FDR) is one way of conceptualizing the rate of type 1 errors in null hypothesis testing when conducting multiple comparisons. FDR-controlling procedures are designed to control the expected proportion of "discoveries" that are false. The problem of multiple testing concerns a group of related null hypotheses *h*^1^, …*h*^*n*^ that are tested simultaneously. In its simplest form, each test yields a summary statistic *z*_*i*_, and the goal is to decide which of the *z*_*i*_ is signals (*h*i = 1) and which are null (*h*i = 0). Solutions to this problem, such as Bonferroni correction, aim to control the family-wise error rate (FWER). An alternative, which has become the dominant approach in many domains of application, is to control the false discovery rate (FDR). Regardless of which error rate they aim to control, however, most existing approaches obey a monotonicity property: if test statistic *z*_*i*_ is declared significant, and *z*_*j*_ is more extreme than *z*_*i*_, then *z*_*j*_ is also declared significant. Extensive simulation evidence shows that, by relaxing the monotonicity property in a data dependent way, FDR regression can improve power while still controlling the global false discovery rate.

The selection of long non-coding RNA was more complex, which was divided into three steps:

#### Size selection

Normally, we defined putative long noncoding RNAs as transcripts that are length ≥ 200bp, and have no or weak protein coding ability [26, 27]. Therefore, we used in house Python scripts to first exclude transcripts smaller than 200bp.

#### Open reading frame filter

Frith et al [28] show that more than 95% of protein-coding genes have ORFs of more than 100 amino acids. Since, we need select transcripts which contained the length of ORF was less than 100bp. To select the transcripts, which were more likely to encode proteins, a Python script was developed to ensure that transcripts that encoded ORFs of 100 or less amino acids were considered as long non-coding RNA candidates.

#### CPC prediction

The Coding Potential Calculator (CPC) [29], which is based on the detection of quality, completeness, and sequence similarity of the ORF to proteins in current protein databases, was utilized to detect putative protein encoding transcripts with default parameters. Only transcripts that did not pass the protein-coding-score test were classified as long noncoding RNAs.

### GO and pathway analysis

GO enrichment analysis [30] was completed by agriGO [31], which was used to compare the biological functions of differentially expressed genes. GO function enrichment was analyzed by Hypergeometric exact test. FDR < 0.05 set as the threshold.

Pathway was analyzed by KOBAS [32–35], which divided into two steps: first, annotating the pathway of differentially expressed genes. E-value of BLAST was 1e-8; second, identifying the significant enrichment pathways. Our differentially expressed genes were clustered by their functions, and identified the pathway processes which affected lots of genes.

## Results

### RNA sequencing and assembly of transcriptome

**Fig 1:**
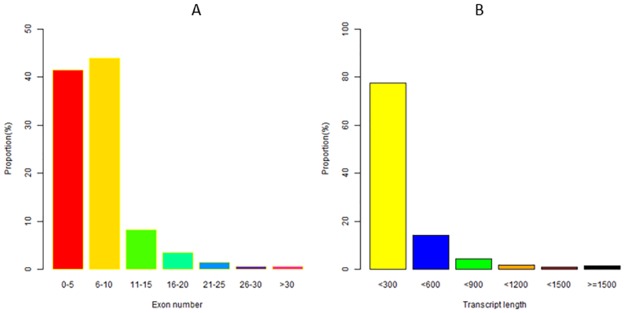
The length and exon number of transcripts.

We obtained a total of 388666 transcripts. There were 16% transcripts in 300nt-600nt and 79% transcripts in 300nt, so we can know that the transcript length mainly concentrated on 300nt-600nt. What’s more, 42% transcripts had less than five exons, and 45% transcripts have 6–10 exons (Fig 1).

### Analysis of gene expression

**Fig 2:**
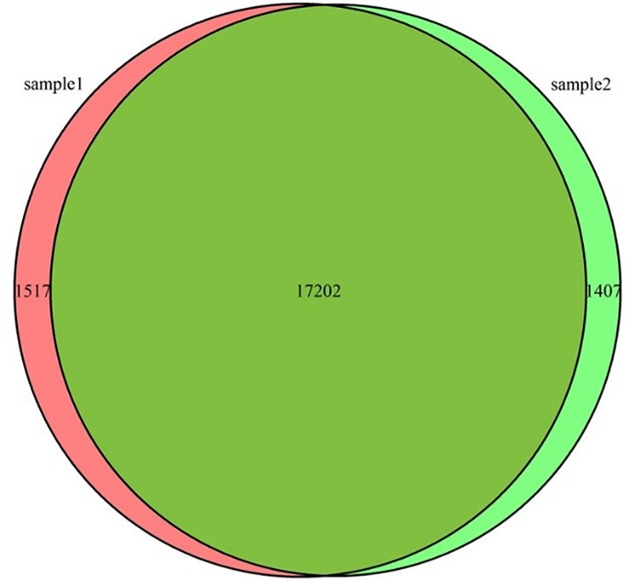
Gene expression map of cultivated and wild tomato. Sample1: cultivated tomato. Sample2: wild tomato.

We classified and counted the expressed genes of cultivated tomato and wild tomato. In addition, we found that there were 18719 expressed genes in cultivated tomato and there were 18609 expressed genes in wild tomato. However, there were 17202 genes in the co-expression of cultivated and wild tomato, which accounted for 91% in cultivated tomato and 92% in wild tomato, respectively. Therefore, it showed that most of genes in cultivated tomato and wild tomato were co-expressed. And genetic similarity is very high (Fig 2).

**Fig 3:**
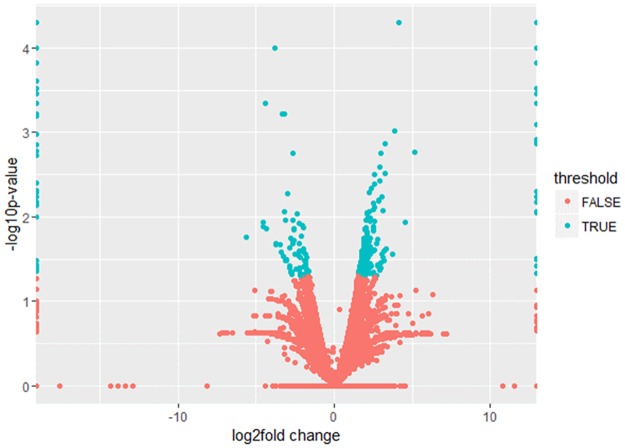
The volcano map of gene expression.

For these genes, we selected differentially expressed genes (Fig 3). We chose p − value and log2fold change as the threshold value. Green was differentially expressed genes and red was normal genes. The differentially expressed genes were mainly distributed in the area of |log2fold change| > 1.

### Differentially expressed genes in cultivated tomato and wild tomato

Through the above methods, we selected some differentially expressed genes. There were 126 up-regulated differentially expressed genes and 87 down-regulated differentially expressed genes, such as (Table 2) and (Table 3). (Table 2) showed a part of up regulated differentially expressed genes, and (Table 3) showed a part of down regulated differentially expressed genes.

**Table 2:** The part of differentially expressed genes (up regulation).

Gene ID	FPKM (sample1)	FPKM (sample2)	P-value	Up/Down	Log2(Fold Change)
Solyc11g071610.1	21.44	0.36	0.00005	Up	5.78470
Solyc00g156980.2	105.69	2.74	5.00E-05	Up	5.27032
Solyc00g012800.1	86.55	3.21	5.00E-05	Up	4.75197
Solyc00g227860.1	20.60	0.92	5.00E-05	Up	4.48968
Solyc00g068980.1	9089.98	485.82	5.00E-05	Up	4.22579
S0lyc00g075440.1	164.77	9.61	5.00E-05	Up	4.09993
Solyc00g068970.2	1503.85	105.92	0.00015	Up	3.82755
Solyc00g009760.2	1149.49	117.40	5.00E-05	Up	3.29152
Solyc00g012430.1	1872.83	193.74	0.00250	Up	3.27299
Solyc00g006690.2	42.50	4.65	0.00440	Up	3.19271

**Table 3:** The part of differentially expressed genes (down regulation).

Gene ID	FPKM (sample1)	FPKM (sample2)	P-value	Up/Down	Log2(Fold Change)
Solyc00g186050.1	10.59	640.38	0.00235	Down	-6.17787
Solyc01g006390.2	1.17	32.32	0.00015	Down	-5.93804
Solyc01g056350.1	3.78	99.96	0.00005	Down	-5.11026
Solyc01g097530.1	11.64	171.65	0.00005	Down	-4.40286
Solyc03g097670.2	283.31	1482.49	0.00005	Down	-2.95376
Solyc02g071820.2	638.25	3383.79	0.0018	Down	-2.50705
Solyc02g077100.2	773.92	3989.27	0.00225	Down	-2.41246
Solyc02g092220.1	1.41	7.42	0.00135	Down	-2.39747
Solyc00g085070.2	1.70	6.10	0.00005	Down	-2.17913
Solyc01g108580.2	4.06	18.59	0.00005	Down	-2.02834

### Differentially expressed genes in different tissues

**Fig 4:**
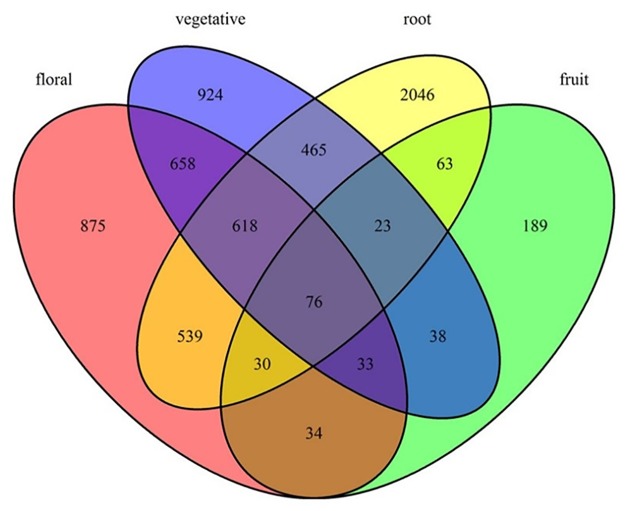
Differentially expressed genes in different tissues.

The distribution of differentially expressed genes was different in every tissue. Our data are from seven tissues, such as floral, fruit, vegetative, root, stem, leaf and seeding. Nevertheless, differentially expressed genes only distributed in four tissues, which were floral, fruit, vegetative and root. There were 76 differentially expressed genes in the four groups together; there were 23 differentially expressed genes in vegetative, root and fruit; there were 618 differentially expressed genes in root, vegetative and floral; there were 30 differentially expressed genes in fruit, root and floral; there were 33 differentially expressed genes in vegetative, floral and fruit. There were 658 differentially expressed genes in vegetative and floral; there were 34 differentially expressed genes in floral and fruit; there were 63 differentially expressed genes in root and fruit; there were 465 differentially expressed genes in vegetative and root. In addition to, there were 875 differentially expressed genes in floral; there were 924 differentially expressed genes in vegetative; there were 2046 differentially expressed genes in root and there were 189 differentially expressed genes in fruit. From (Fig 4), we can clearly see the differentially expressed genes in different tissues.

**Fig 5:**
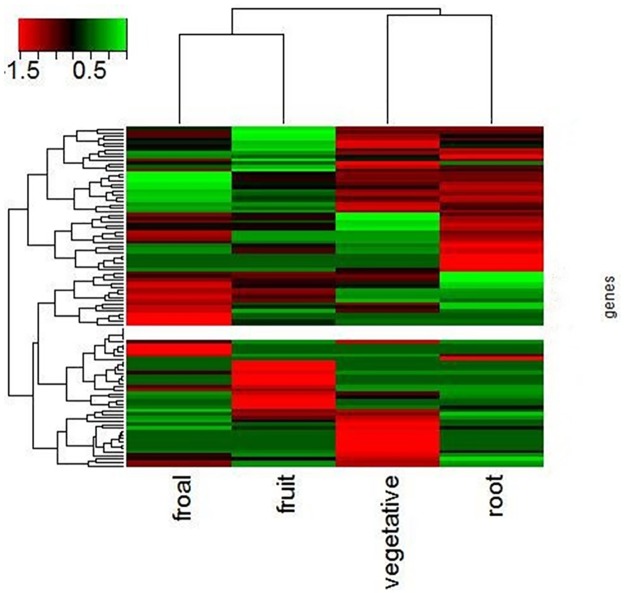
Clusters of expression levels of the candidate genes in the four tissues.

We also analyzed the expression level of differentially expressed genes, as follow in (Fig 5). From (Fig 5), we can know the expression level of floral was highest in the four tissues. The second one was root, and the expression level of vegetative and fruit was lower than others. Meanwhile, we can clearly see the expression levels of differentially expressed genes in different tissues. The clustering of differentially expressed genes in different tissues also reflects the difference between domesticated and wild tomato. The difference in clustering may be closely related to drought resistance, salt tolerance and fruit nutrient composition of tomato.

### Function analysis and annotation of differentially expressed genes

**Fig 6:**
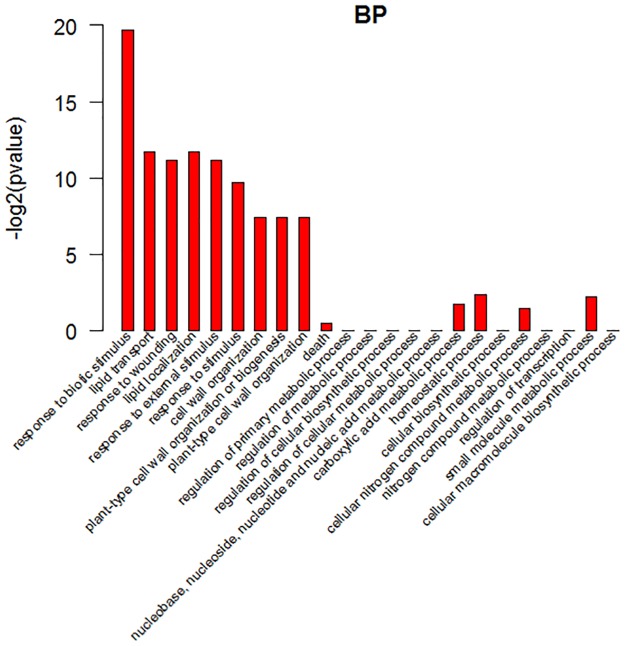
GO analysis of differentially expressed genes. Biological process ontologies of DEGs shared by cultivated tomato and wild tomato. In the ontology, the first 23 enriched terms were listed.

**Fig 7:**
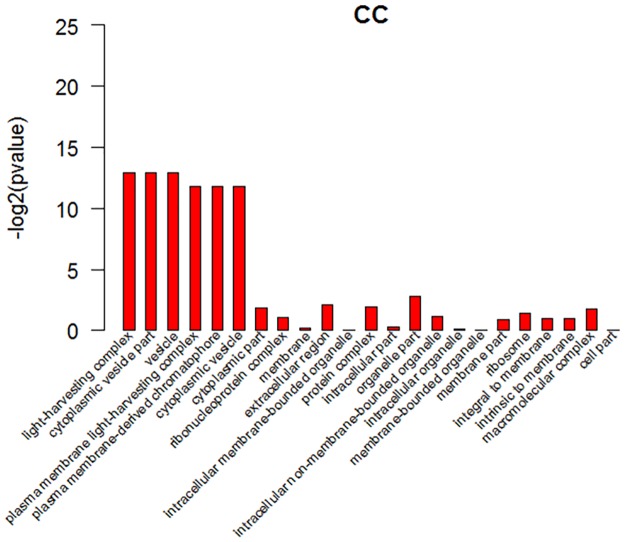
GO analysis of differentially expressed genes. Cellular component ontologies of DEGs shared by cultivated tomato and wild tomato. In the ontology, the first 23 enriched terms were listed.

**Fig 8:**
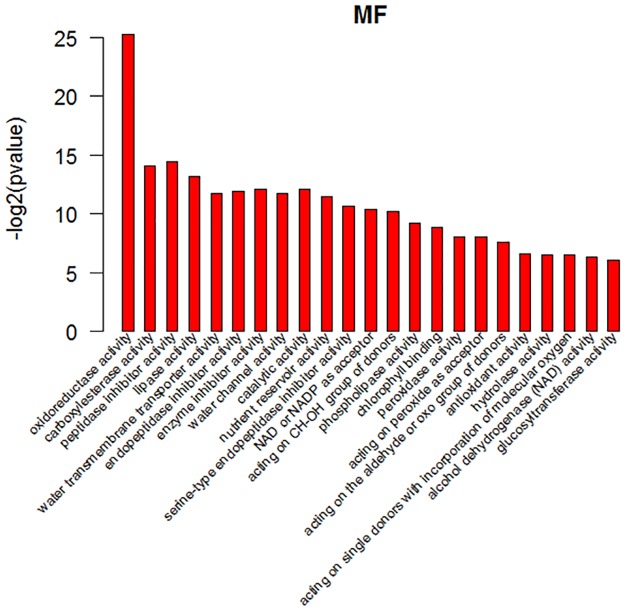
GO analysis of differentially expressed genes. Molecular function ontologies of DEGs shared by cultivated tomato and wild tomato. In the ontology, the first 23 enriched terms were listed.

GO analysis showed that the gene annotations were mainly in three aspects: biological processes (BP), molecular function (MF), and cell fractions (CC). In the biological process, the differentially expressed genes were mainly enriched in response to biotic stimulus, lipid transport, response to wounding, response to extremal stimulus, response to stimulus and so on, as shown in (Fig 6) in detail. In the cell components, the differentially expressed genes were mainly enriched in light-harvesting complex, cytoplasmic vesicle part, vesicle, plasma membrane light-harvesting complex, plasma membrane-derived chromophore, cytoplasmic vesicle and so on, as shown in (Fig 7) in detail. In the molecular function, the differentially expressed genes were mainly enriched in oxidoreductase activity, carboxylesterase activity, peptidase inhibitor activity, lipase activity, water transmembrane transporter activity, endopeptidase inhibitor activity, enzyme inhibitor activity, water channel activity, catalytic activity, nutrient reservoir activity, serine-type endopeptidase inhibitor activity, oxidoreductase activity and acting on the CH-OH group of donors, as shown in (Fig 8) in detail. These data showed that the three class of differentially expressed genes exhibited different GO functions, implying difference between the two different samples.

### Pathway analysis of differentially expressed genes

Pathway analysis showed the differences of gene expression from another perspective. This analysis was similar to GO enrichment analysis.

**Fig 9:**
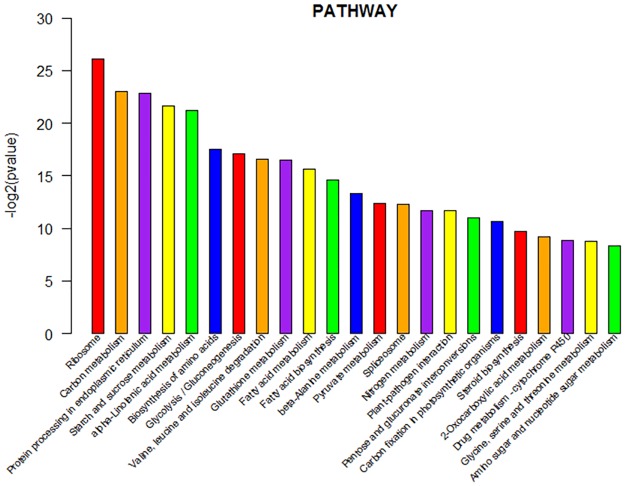
KEGG analysis of differentially expressed genes. Pathway prediction of DEGs shared by cultivated tomato and wild tomato. In each category, the first 23 enriched terms were listed.

KEGG pathway analysis which was represented in the (Fig 9) illustrated the disparities of the three DEG categories from another perspective. The terms enriched in DEGs were Carbon metabolism, protein processing in endoplasmic reticulum, starch and sucrose metabolism, alpha-linolenic add metabolism, biosynthesis of amino acids, glycolysis / gluconeogenesis, valine, leucine and isoleucine degradation, glutathione metabolism and so on. The difference of domesticated tomato and wild tomato mainly was showed in the above biological pathway. These pathways may be important factors that affected the heat resistance, salt tolerance and so on.

### Functional annotation of differentially expressed genes in different tissues

In different tissues, the distribution of differentially expressed genes was not identical. DGEs mainly distributed in floral, fruit, vegetative and root. The others have few differentially expressed genes. Therefore, the significant GO enriched analysis of differentially expressed genes showed as follow.

#### Differentially expressed genes belonging to floral group

**Fig 10:**
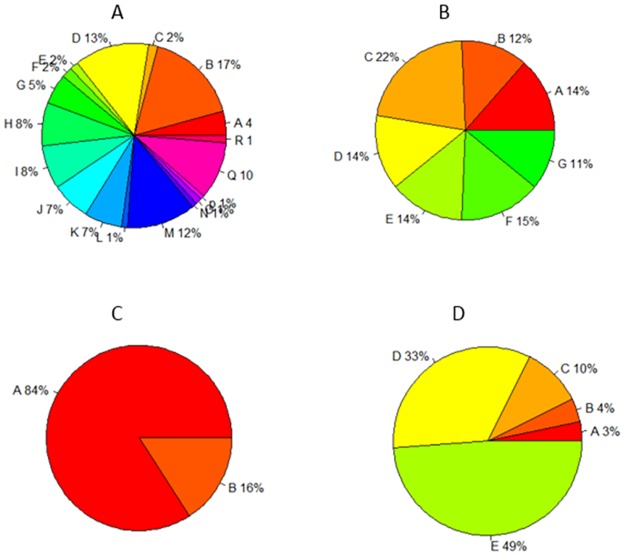
GO analysis of differentially expressed genes in each tissue. BP, MF and CC ontologies of DEGs shared by cultivated tomato and wild tomato.

GO enrichment terms in the floral group related to CC and MF ontologies. In the cell components, DEGs were mainly enriched in cytoplasm (13%), macromolecular complex (12%), cytoplasmic part (10%), intracellular non-membrane bounded organelle (8%) and non-membrane-bounded organelle (8%), as shown in (Fig 10A). In the molecular function, DGEs were mainly enriched in oxidoreductase activity (17%) and structural molecule activity (5%), as shown in (Fig 10A).

#### Differentially expressed genes belonging to fruit group

GO enrichment terms in the fruit group related to BP ontologies. In the biology process, DEGs were mainly enriched in small molecule metabolic process (22%), organic acid metabolic process (15%), carboxylic acid metabolic process (14%), oxoacid metabolic process (14%) and cellular ketone metabolic process (13%), as shown in (Fig 10B).

#### Differentially expressed genes belonging to vegetative group

GO enrichment terms in the vegetative group related to MF ontologies. In the molecular function, DEGs were mainly enriched in catalytic activity (84%) and oxidoreductase activity (16%), as shown in (Fig 10C).

#### Differential expressed genes belonging to root group

GO enrichment terms in the root group related to MF ontologies. In the molecular function, DEGs were mainly enriched in catalytic activity (49%), protein binding (33%) and oxidoreductase activity (10%) as shown (Fig 10D) in detail.

### The analysis of differentially expressed long non-coding RNAs

We predicted 554 differentially expressed long non-coding RNAs and 426 coding RNAs, as shown in the following (Table 4).

**Table 4:** A part list of noncoding RNAs and coding RNAs.

RNA id	Exon number	state	coding potential score	Length
Solyc00g010931.1.1	1	noncoding	-1.42749	260
Solyc00g068990.1.1	1	noncoding	-1.41087	266
Solyc00g272810.1.1	1	noncoding	-1.3848	727
Solyc00g009650.1.1	1	noncoding	-1.37232	319
Solyc00g006710.1.1	1	noncoding	-1.36861	251
Solyc00g184350.2.1	1	noncoding	-1.32888	881
Solyc00g227860.1.1	1	noncoding	-1.31422	1414
Solyc00g009661.2.1	1	noncoding	-1.26988	272
Solyc01g009750.2.1	1	noncoding	-1.11849	4451
Solyc01g068020.2.1	1	noncoding	-1.32972	2798
Solyc01g056650.1.1	2	coding	1.20957	4491
Solyc00g282510.1.1	2	coding	6.51034	2180
Solyc01g091050.2.1	2	coding	2.97256	12246
Solyc01g007920.2.1	3	coding	1.89034	3305
Solyc01g008100.2.1	3	coding	2.00741	4236
Solyc00g098560.2.1	4	coding	1.95225	1211
Solyc01g081190.2.1	4	coding	2.22955	1760
Solyc01g081190.2.1	4	coding	2.22955	1760
Solyc01g068430.1.1	5	coding	1.60831	3216
Solyc01g008710.2.1	6	coding	3.43996	3051

From (Table 4), we can see the number of long noncoding RNAs’ exons was fewer than coding RNAs. The number of long noncoding RNAs’ exons was always 1 or 2. However, the number of mRNAs’ exons was ≥2, and it was 5 to 6 currently. Therefore, the average number of exons in long noncoding RNAs was only 1/3 of the average number of exons in the mRNAs. In addition, the percentage of long non-coding RNAs which contained one exon was 50%. And, the percentage of mRNAs which contained single exon was very small. In addition, the length of long non-coding RNAs was much shorter than that of mRNAs. The length of long non-coding RNAs was almost 2000bp. The most of was shorter than 2000bp and a few was longer than 2000bp. The length of mRNAs was ≥3000bp, and a few was <3000bp. So the length of long non-coding RNAs was much shorter than that of mRNAs. Comparing with mRNAs, the number of long noncoding RNAs’ exons was fewer and the length of long noncoding RNAs was much shorter.

**Fig 11:**
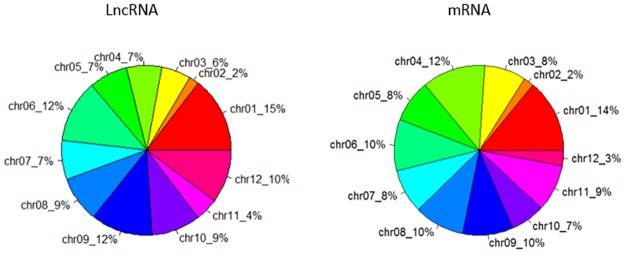
Location distribution of lncRNA and mRNA.

**Fig 12:**
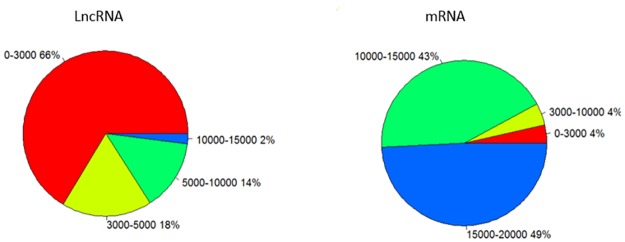
Length distribution of lncRNA and mRNA.

**Fig 13:**
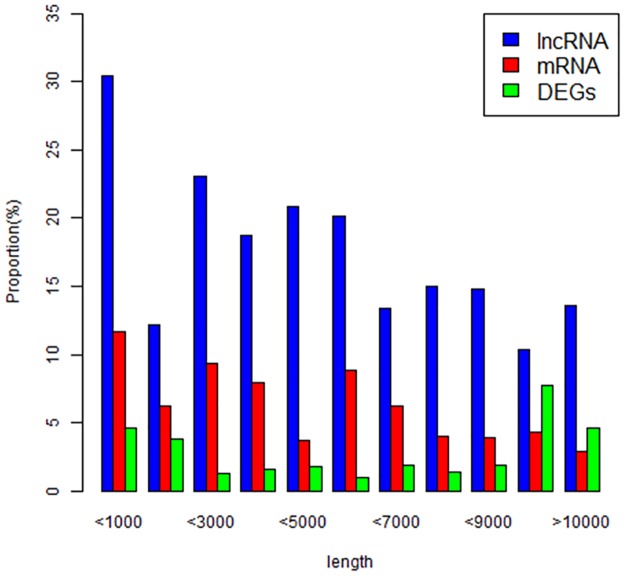
Transcript length. (differentially expressed long non-coding RNA, mRNA and differentially expressed genes).

We can also see that long noncoding RNAs were mainly distributed on chromosome 1, 6, 9 and 10, while mRNAs were mainly distributed on chromosome 1, 6, 8 and 9 (Fig 11). The distribution of length was different between long non-coding RNAs and mRNAs (Fig 12). The majority of long non-coding RNAs’ length (66%) was shorter than 3000bp. The remaining 18% of long noncoding RNAs’ length was shorter than 5000bp. The others were longer than 5000bp. While only 4% mRNAs’ length was shorter than 3000bp. The remaining 43% of mRNAs’ length was longer than 10000bp. The majority of mRNAs’ length (49%) was longer 15000bp (Fig 13). In addition to, we also made a comparison of lncRNAs, mRNAs and DGEs in transcripts length (Fig 13). In the range of 0-1000bp, long non-coding RNAs’ percentage was 30%, mRNAs’ percentage was 11% and differentially expressed genes’ percentage was 5%. In the range of 1000-3000bp, long non-coding RNAs’ percentage was 25%, mRNAs’ percentage was 10% and differentially expressed genes’ percentage was 4%. In the range of 9000-10000bp, long non-coding RNAs’ percentage was 12%, mRNAs’ percentage was 5%, differentially expressed genes’ percentage was 10%. Overall, as the growth of the transcript’s length, the percentage of long non-coding RNAs is smaller and smaller.

**Fig 14:**
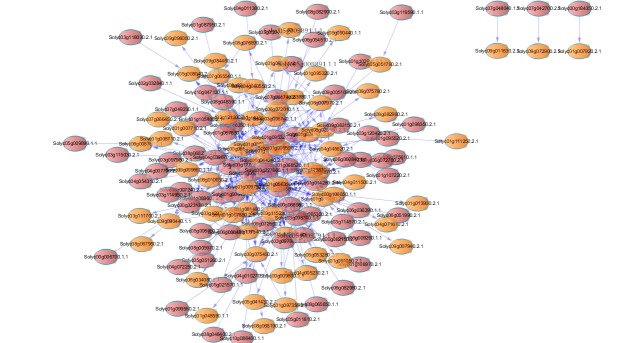
Co-expression network of between lncRNAs and mRNAs.

In addition, we constructed a co-expression network of lncRNA and mRNA to predict the concrete functions of lncRNAs (Fig 14). In (Fig 14), red represents mRNA, and orange represents lncRNA. Based on the co-expression network of lncRNA and mRNA, we predicted the lncRNA target gene (Table 5). From (Table 5), we can know that GO annotation of these target genes. The function of the target gene is mainly distributed in response to biotic stimulus, peptidase inhibitor activity and so on. LncRNA plays an important role in regulating the expression of the genes.

**Table 5:** Functional annotation of lncRNA target gene.

Target Gene	Feature	p-value	FDR
Solyc08g023660.2.1	response to biotic stimulus	2.10E-05	0.0014
Solyc03g098740.1.1	peptidase inhibitor activity	3.50E-05	0.0052
Solyc06g071290.2.1	oxidoreductase activity	4.40E-05	0.0052
Solyc03g098740.1.1	enzyme inhibitor activity	5.40E-05	0.0052
Solyc08g029000.2.1	oxidoreductase activity, acting on single donors with incorporation of molecular oxygen, incorporation of two atoms of oxygen	0.00029	0.017
Solyc09g072560.2.1	nutrient reservoir activity	0.0003	0.017
Solyc03g098740.1.1	enzyme regulator activity	0.00041	0.017
Solyc08g029000.2.1	oxidoreductase activity, acting on single donors with incorporation of molecular oxygen	0.00042	0.017
Solyc07g054720.1.1	dioxygenase activity	0.00047	0.017
Solyc07g007260.2.1	endopeptidase inhibitor activity	0.00068	0.022

## Discussion

In this study, the transcriptome of cultivated tomato was sequenced by Illumina high throughput sequencing and compared with the transcriptome of wild tomato, including differentially expressed genes and long non-coding RNAs. Our data reflects the differences between cultivated tomato and wild tomato and offers tomato’s basic transcriptional information in future tomato research. As DEGs results showed, there are 87 down-regulated genes and 126 up-regulated genes in tomato. It could be deduced that the expression level of DEGs in cultivated tomato and wild tomato is quite high. DEGs of up regulated or down regulated in both cultivated tomato and wild tomato were verified. We performed functional analysis and distribution analysis of differentially expressed genes. Through the enriched analysis of GO and Pathway, we mainly analyzed the functions and biological pathways of these differentially expressed genes, including the bioactivity response, lipid transport, light absorption complexity, cytoplasmic vesicle, plasma membrane light-gathering complexity, carbon metabolism, protein processing, starch and sucrose metabolism, amino acid biosynthesis. DGEs mainly distributed in floral, fruit, vegetative and root and the others have few differentially expressed genes. Differentially expressed genes are distributed in different tissues, and GO enrichment is also different in every tissue. These features reflect the phenotypic difference between domesticated and wild tomato. There is difference in different tissues for differentially expressed genes, such as distribution, function and so on. In the root and vegetative tissues, differentially expressed genes are most of all. In the root group, differentially expressed genes were mainly enriched in catalytic activity (49%), protein binding (33%) and oxidoreductase activity (10%). In the vegetative group, differentially expressed genes were mainly enriched in catalytic activity (84%) and oxidoreductase activity (16%). This finding plays an important role in improving the resistance of domesticated tomatoes. In consequence, wild tomato has better salt resistance and drought resistance, and cultivated tomato’s fruit is bigger and has more nutrition [36]. These features of domesticated tomato and wild tomato may be related to differentially expressed genes. The results above provide a scientific basis for tomato’s cultivation and study in future.

In addition, long non-coding RNAs are critical in regulating post-transcriptional regulation of RNA silencing and gene expression. We analyzed basic characteristic of long non-coding RNA, and made a series of comparison with mRNA. These long noncoding RNAs also tend to be shorter and to have fewer introns than protein-coding transcripts. The length of long non-coding RNA is shorter than coding RNA and the number of long noncoding RNA’s exon is less than coding RNA. Meanwhile, we also drawn a co-expression network between long non-coding RNA and mRNA. There is a correlation between long non-coding RNA and mRNA. Long non-coding RNA regulated mRNA’s expression. Long non-coding RNA may also be a key factor in the phenotypic variation of domestic and wild tomatoes.

As discussed above, cultivated tomato could be different from wild tomato based on DEGs and differentially expressed long noncoding RNAs. Disparities which are also found between cultivated tomato and wild tomato are used in many studies. To sum up, the study has compared the transcriptome of cultivated tomato and wild tomato based on data generated from RNA-sequencing. Analysis of DEGs and differentially expressed long noncoding RNAs has revealed important features of cultivated tomato and wild tomato. To better utilize tomato’s transcriptional information and to verify the functional mechanisms of DEGs and long non-coding RNAs, it is important to further research.

## References

[1] Correia AF, Loro AC, Zanatta S, Spoto MH, Vieira TM. Effect of Temperature, Time, and Material Thickness on the Dehydration Process of Tomato. Int J Food Sci 2015;970724 10.1155/2015/970724 26904666 PMC4745559

[2] Ghosh S, Singh UK, Meli VS, Kumar V, Kumar A, Irfan M, et al Induction of senescence and identification of differentially expressed genes in tomato in response to monoterpene. PLoS One 2013;8(9):e76029 10.1371/journal.pone.0076029 24098759 PMC3786903

[3] Li P, Yin F, Song L, Zheng X. Alleviation of chilling injury in tomato fruit by exogenous application of oxalic acid. Food Chem 2016;202:125–32. 10.1016/j.foodchem.2016.01.142 26920276

[4] Sacilik K. The thin-layer modelling of tomato drying process. Agriculturae Conspectus Scientifius 2007;72(4):349.

[5] Souza JS. Estudo da desidratacao do tomato (Lycopersicon esculentum Mill) empedacos compre-tratamento osmotico [Dissertacao Programa de pos-graduacao em Engenharia Quımica]. Universidade Federal do Rio Grande do Norte 2002.

[6] George S, Tourniaire F, Gautier H, Goupy P, Rock E, Caris-Veyrat C. Changes in the contents of carotenoids, phenolic compounds and vitamin C during technical processing and lyophilisation of red and yellow tomatoes. Food Chem 2011;1624:1633.

[7] Shi J, Le Maguer M. Lycopene in tomatoes: chemical and physical properties affected by food processing. Crit Rev Biotechnol 2000 1;20(4):293–334. 10.1080/07388550091144212 11192026

[8] Khachik F, Carvalho L, Bernstein PS, Muir GJ, Zhao DY, Katz B. Chemistry, distribution, and metabolism of tomato carotenoids and their impact on human health. Exp Biol Med 2002;227(10):851. 10.1177/153537020222701002 12424324

[9] Doebley JF, Gaut BS, Smith BD. The molecular genetics of crop domestication. Cell 2006 12 29;127(7):1309 10.1016/j.cell.2006.12.006 17190597

[10] Izawa T, Konishi S, Shomura A, Yano M. DNA changes tell us about rice domestication. Curr Opin Plant Biol 2009 4;12(2):185 10.1016/j.pbi.2009.01.004 19185529

[11] Paran I, Knaap E. Genetic and molecular regulation of fruit and plant domestication traits in tomato and pepper. J Exp Bot 2007;58(14):3841 10.1093/jxb/erm257 18037678

[12] Sato Y, Tabata S, Hirakawa H, Asamizu E, Shirasawa K, Isobe S, et al The tomato genome sequence provides insights into fleshy fruit evolution. Nature 2012 5 31;485(7400):635–41. 10.1038/nature11119 22660326 PMC3378239

[13] Tal M, Heikin H, Dehan K. Salt tolerance in the wild relatives of the cultured tomato:responses of callus tissues of Lycopersicon esculentum and Solanum pennellii to high salinity. Zpflanzenphysiol 1978;86:9.

[14] Chen H, Chen X, Chai X, Qiu Y, Gong C, Zhang Z, et al Effects of low temperature on mRNA and small RNA transcriptomes in Solanum lycopersicoides leaf revealed by RNA-Seq. Biochem Biophys Res Commun 2015;464(3):768 10.1016/j.bbrc.2015.07.029 26187671

[15] Koenig D, Jimenez-Gomez JM, Kimura S, Fulop D, Chitwood DH, Headland LR, et al Comparative transcriptomics reveals patterns of selection in domesticated and wild tomato. Proc Natl Acad Sci U S A 2013;110(28):E2655–62. 10.1073/pnas.1309606110 23803858 PMC3710864

[16] Li KQ, Xu XY, Huang XS. Identification of Differentially Expressed Genes Related to Dehydration Resistance in a Highly Drought-Tolerant Pear, Pyrus betulaefolia, as through RNA-Seq. PLoS One 2016;11(2):e0149352 10.1371/journal.pone.0149352 26900681 PMC4762547

[17] Ruiu F, Picarella ME, Imanishi S, Mazzucato A. A transcriptomic approach to identify regulatory genes involved in fruit set of wild-type and parthenocarpic tomato genotypes. Plant Mol Biol 2015;89(3):263–78. 10.1007/s11103-015-0367-1 26319515

[18] Koenig D, Jimenez-Gomez JM, Kimura S, Fulop D, Chitwood DH, Headland LR, et al Comparative transcriptomics reveals patterns of selection in domesticated and wild tomato. Proc Natl Acad Sci U S A 2013;110(28):E2655–62. 10.1073/pnas.1309606110 23803858 PMC3710864

[19] The sequence read archive. http://www.ncbi.nlm.nih.gov/Traces/sra/.

[20] Kroll KW, Mokaram NE, Pelletier AR, Frankhouser DE, Westphal MS, Stump PA, et al Quality Control for RNA-Seq (QuaCRS): An Integrated Quality Control Pipeline. Cancer inform 2014;13(Suppl 3):7 10.4137/CIN.S14022 25368506 PMC4214596

[21] Kumar S, Vo AD, Qin F, Li H. Comparative assessment of methods for the fusion transcripts detection from RNA-Seq data. Sci Rep 2016;6:21597 10.1038/srep21597 26862001 PMC4748267

[22] Mutz KO, Heilkenbrinker A, Lonne M, Walter JG, Stahl F. Transcriptome analysis using next-generation sequencing. CURR OPIN BIOTECH 2013;24(1):22–30. 10.1016/j.copbio.2012.09.004 23020966

[23] Wang Z, Gerstein M, Snyder M. RNA-Seq: a revolutionary tool for transcriptomics. Nat Rev Genet 2009 1;10(1):57 10.1038/nrg2484 19015660 PMC2949280

[24] Trapnell C, Pachter L, Salzberg SL. TopHat: discovering splice junctions with RNA-Seq. Bioinformatics 2009 5 1;25(9):1105–11. 10.1093/bioinformatics/btp120 19289445 PMC2672628

[25] Trapnell C, Roberts A, Goff L, Pertea G, Kim D, Kelley DR, et al Differential gene and transcript expression analysis of RNA-seq experiments with TopHat and Cufflinks. Nat Protoc 2012 3;7(3):562 10.1038/nprot.2012.016 22383036 PMC3334321

[26] Shoaib M, Gul KMR, Jianwen S, Sadia M, Yuyang Z, Zhibiao Y, et al Genome-wide identification, characterization and expression analysis of calmodulin-like (CML) proteins in tomato (Solanum lycopersicum). Plant Physiol Biochem 2016;102:167 10.1016/j.plaphy.2016.02.020 26949025

[27] Mortazavi A, Williams BA, McCue K, Schaeffer L, Wold B. Mapping and quantifying mammalian transcriptomes by RNA-Seq. Nat Methods 2008 7;5(7):621 10.1038/nmeth.1226 18516045 PMC13303166

[28] Pang KC, Grimmond SM. The abundance of short proteins in the mammalian proteome. PLoS genetics 2006;2:e52 10.1371/journal.pgen.0020052 16683031 PMC1449894

[29] Kong L, Zhang Y, Ye ZQ, Liu XQ, Zhao SQ, Wei L, et al CPC: assess the protein-coding potential of transcripts using sequence features and support vector machine. Nucleic Acids Res 2007 7;35(Web Server issue):W345–9. 10.1093/nar/gkm391 17631615 PMC1933232

[30] Xu X, Wang X, Fu B, Meng L, Lang B. Differentially expressed genes and microRNAs in bladder carcinoma cell line 5637 and T24 detected by RNA sequencing. Int J Clin Exp Pathol 2015;8(10):2625. PMC4680402 26722457

[31] Du Z, Zhou X, Ling Y, Zhang Z, Su Z. agriGO: a GO analysis toolkit for the agricultural community. Nucleic Acids Res 2010;38:W70. 10.1093/nar/gkq310 PMC2896167 20435677

[32] Kanehisa M, Sato Y, Kawashima M, Furumichi M, Tanabe M. KEGG as a reference resource for gene and protein annotation. Nucleic Acids Res 2016 1 4;44(D1):D457 10.1093/nar/gkv1070 26476454 PMC4702792

[33] Xie C, Mao X, Huang J, Ding Y, Wu J, Dong S, et al KOBAS 2.0: a web server for annotation and identification of enriched pathways and diseases. Nucleic Acids Res 2011;39:w322. 10.1093/nar/gkr483 PMC3125809 21715386

[34] Wu J, Mao X, Cai T, Luo J, Wei L. KOBAS server: a web-based platform for automated annotation and pathway identification. Nucleic Acids Res 2006;34:W724. 10.1093/nar/gkl167 PMC1538915 16845106

[35] Mao X, Cai T, Olyarchuk JG, Wei L. Automated genome annotation and pathway identification using the KEGG Orthology (KO) as a controlled vocabulary. Bioinformatics 2005;21:3793. 10.1093/bioinformatics/bti430 15817693

[36] Liu H, Ouyang B, Zhang J, Wang T, Li H, Zhang Y, et al Differential modulation of photosynthesis, signaling, and transcriptional regulation between tolerant and sensitive tomato genotypes under cold stress. PLoS One 2012;7(11):e50785 10.1371/journal.pone.0050785 23226384 PMC3511270

